# Impact of Food Rations and Supplements on Micronutrient Status by Trimester of Pregnancy: Cross-Sectional Studies in the Maela Refugee Camp in Thailand

**DOI:** 10.3390/nu8020066

**Published:** 2016-01-26

**Authors:** Wolfgang Stuetz, Verena I. Carrara, Rose Mc Gready, Sue J. Lee, Kanlaya Sriprawat, Basi Po, Borimas Hanboonkunupakarn, Tilman Grune, Hans K. Biesalski, François H. Nosten

**Affiliations:** 1 Institute of Biological Chemistry and Nutrition, University of Hohenheim, Stuttgart 70599, Germany; biesal@uni-hohenheim.de; 2 Shoklo Malaria Research Unit, Mahidol-Oxford Tropical Medicine Research Unit, Faculty of Tropical Medicine, Mahidol University, Mae Sot 63110, Thailand; verena@shoklo-unit.com (V.I.C.); rose@shoklo-unit.com (R.M.G.); poo@shoklo-unit.com (K.S.); smru@tropmedres.ac (B.P.); francois@tropmedres.ac (F.H.N.); 3 Centre for Tropical Medicine, Nuffield Department of Medicine, University of Oxford, Oxford OX3 7BN, UK; sue@tropmedres.ac; 4 Mahidol-Oxford Tropical Medicine Research Unit, Faculty of Tropical Medicine, Mahidol University, Bangkok 10400, Thailand; Borimas@tropmedres.ac; 5 German Institute of Human Nutrition, Potsdam-Rehbruecke, 14558 Nuthetal, Germany; scientific.director@dife.de

**Keywords:** micronutrients, pregnancy, refugee, iron status, zinc, tocopherol, folic acid, thiamine, retinol, β-carotene

## Abstract

Micronutrient fortified flour (MFF), supplementary food rations and micronutrient (MN) supplements may prevent deficiencies among pregnant women. Objectives of cross-sectional surveys in 2004 (*n* = 533) and 2006 (*n* = 515) were to assess the impact of new food rations (flour, oil) and supplements on MN status by trimester of pregnancy in the Maela refugee camp. Hemoglobin, iron status, zinc, retinol, β-carotene and tryptophan decreased, while α-/γ-tocopherol and 5-methyltetrahydrofolate (5-MTHF) increased from first to third trimester. In 2006, mean zinc and α-tocopherol for each trimester was significantly higher than in 2004. The weeks of supplemented thiamine and folic acid were positively correlated with thiamine diphosphate (TDP) and 5-MTHF, but not for ferrous sulfate as iron deficiency was observed in 38.5% of third-trimester women. Frequent consumption of fish paste and owning a garden or animal were associated with significantly higher iron status, retinol, β-carotene, and 5-MTHF. In conclusion, MFF and supplementary oil were most likely to explain improved zinc and α-tocopherol status, while thiamine and folate supplements ensured high TDP and 5-MTHF in late pregnancy. MN supplements, MN-rich staple food, small gardens, and programs to improve iron compliance are promising strategies to prevent MN deficiencies during pregnancy in vulnerable populations.

## 1. Introduction

Securing adequate maternal nutrition with essential micronutrients (MNs) poses a difficult challenge in acute and protracted refugee settings [1]. The Maela refugee camp remains the largest border camp in Western Thailand with up to 50,000 inhabitants of predominantly Karen ethnic origin from neighboring Myanmar [2]. Nutrition in this setting relies mainly on the provided food basket consisting of rice, split mung beans, fermented fish, iodized salt, soybean oil and dried chilies [3]. Pregnant women are routinely provided with additional food rations (e.g., mung beans, fish) and MN supplements (iron, folate, thiamine) to prevent MN deficiencies [4]. In July 2004, MN fortified flour (MFF) was introduced as a supplementary food ration to all inhabitants in the Maela camp [5]; in February 2005, an additional oil ration for pregnant and post-partum women was provided in the Shoklo Malaria Research Unit (SMRU) antenatal clinics (ANC).

Blood MNs and hemoglobin concentration in pregnant women can be modulated by food rations, the provision of MN supplements, the availability of seasonal MN-rich food, and also with normal physiological adaptations that occur during gestation such as plasma volume expansion [6,7,8,9,10,11,12,13]. The aim of the present study was to evaluate the impact of the dietary changes between 2004 and 2006 on micronutrient status (hemoglobin, iron status, retinol, α-tocopherol, zinc and thiamine amongst other MNs) in each trimester of pregnancy.

## 2. Materials and Methods

### 2.1. Study Population and Field Procedure

This study was conducted at the SMRU ANC in the Maela camp, 50 km north of Mae Sot on the Thai Myanmar border, according to the guidelines in the Declaration of Helsinki. The study was approved by the Ethics Committee of the Faculty of Tropical Medicine of Mahidol University (TM-IRB 04/2004) in Thailand and the Oxford Tropical Research Ethics Committee, University of Oxford (OXTREC 009-04), UK, and was registered at the German Clinical Trials Register (http://www.drks.de/DRKS00007736).

**Figure 1:**
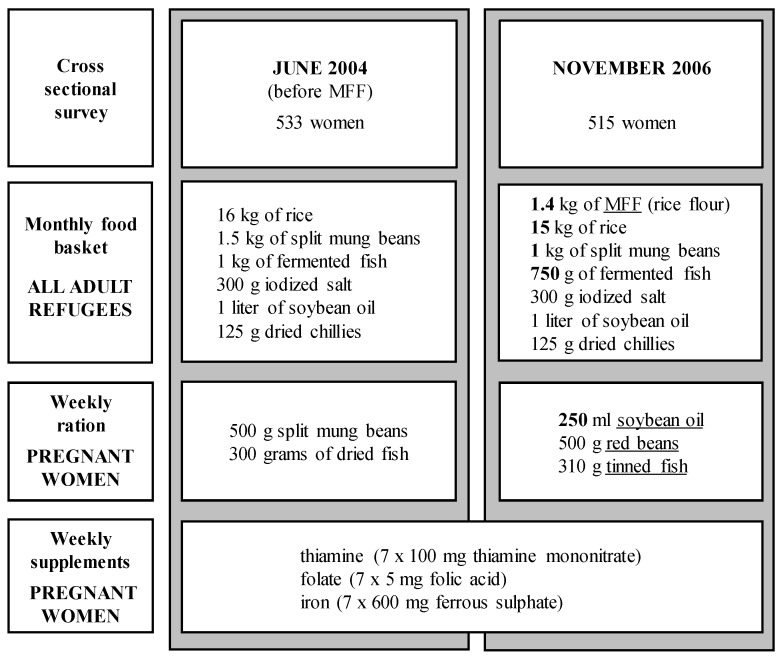
Food rations and supplements by survey. Changes indicated by underline; grey boxes indicate cross-sectional surveys.

A total of 1048 pregnant women (1st to 3rd trimester) were enrolled in two sequential cross-sectional studies at the antenatal clinics in the Maela camp. The first survey was conducted in June 2004, immediately prior to the introduction of MFF, and the second survey in November 2006, by which time all enrolled pregnancies would have received a minimum of three months pre-conceptual exposure to the MFF (Figure 1). All women gave written consent for a blood sample, which was collected only once in the pregnancy. Exclusion criteria included severe anemia, not receiving the refugee food ration, living outside the camp, or planning to deliver elsewhere.

### 2.2. Gestational Age and Maternal Anthropometry

Gestational age (GA) was estimated by ultrasound measurements at the first antenatal consultation (between 8 and 12 weeks) and again around 18 weeks (17 to 22 weeks); quality control of the ultrasound dating has been previously reported [14]. Over 90% of the pregnant women in the Maela camp attend weekly ANC until delivery. The definition of trimesters are as follows: trimester one: <14 weeks; trimester two: 14 to <28 weeks; trimester three: ≥28 weeks of pregnancy.

Maternal weight and height were measured at the first antenatal consultation in the SMRU clinics and at the time of sampling, and used to calculate body mass index (BMI). Normal values of BMI for the Asian population as proposed by the WHO range between 18.5 and 23.0 kg/m^2^ [15]. Therefore, BMI <18.5 kg/m^2^, ≥23 kg/m^2^ and ≥27.5 kg/m^2^ were used as cut-offs, indicating underweight, overweight and obesity, respectively.

### 2.3. Dietary Changes

In June 2004, the standard monthly food basket for adult refugees in 2004 included 16 kg of rice, 1.5 kg of split mung beans, 1 kg of fermented fish, 300 g iodized salt, 1 L of soybean oil and 125 g of dried chilies. In July 2004, MFF (whole wheat flour) with basic vitamins and minerals was introduced for all inhabitants of the Maela camp [5]. Each adult was provided with 1.4 kg of MFF per month and the rations of rice and mung bean were reduced to 15 and 1 kg, respectively. In March 2005, whole-wheat flour was replaced by the rice-flour-based “Asia Mix” and fermented fish by 750 g of cleaned sea fish (Figure 1). The estimated daily intakes are based on a one-to-one consumption of distributed rations. As the household ration is distributed on a monthly basis intra-household redistributions may well take place. Seasonal fruits and vegetables could supplement the monthly food basket, household expenditure permitting.

The weekly supplementary ration for pregnant women in June 2004 included 500 g split mung beans and 300 g of dried fish. In February 2005, an additional weekly ration of soybean oil (250 mL) was provided, and split mung beans and dried fish were replaced by red beans and tinned fish. During both cross-sectional studies, weekly MN supplements for pregnant women consisted of thiamine (7 × 100 mg thiamine mononitrate), folate (7 × 5 mg folic acid) and iron (7 × 600 mg ferrous sulfate) and were provided weekly during ANC consultations (Figure 1).

### 2.4. Laboratory Investigations

Capillary blood for hematocrit measurement was collected on-site in the Maela camp and used to derive hemoglobin levels [16]. Anemia was defined as hemoglobin <110 g/L in 1st and 3rd and <105 g/L in 2nd trimester [17]. Non-fasting EDTA-whole blood (5 mL) and whole blood for serum was collected between 10:00 a.m. and 12:00 midday by venipuncture into vacutainers. Whole blood and serum were portioned into eppendorf tubes and frozen at −20 °C before transportation to the SMRU office in Mae Sot, where they were stored at −80 °C. Samples were then sent (on dry ice) to the University of Hohenheim in Stuttgart, Germany for the analysis of iron status, infection markers, fat-soluble vitamins and trace elements of serum and thiamine diphosphate in whole blood [5].

In brief, serum ferritin and soluble transferrin receptor (sTfR), as indicators for iron status, and the acute phase proteins C-reactive protein (CRP) and α-1 glycoprotein (AGP), were measured by ELISA. Iron deficiency (ID) was defined as ferritin <12 µg/L or sTfR >8.5 mg/L [18]; CRP >5 μg/L and AGP >1 g/L were used as indicators for an acute phase response by infection or inflammation [19]. Zinc and copper in serum were analyzed by inductively coupled plasma mass spectrometry (ICP-MS). Serum zinc <0.56 mg/L in trimester 1 and <0.50 mg/L in trimesters 2 and 3 was used to indicate zinc deficiency [20]. Retinol, α-/γ-tocopherol, and α-/β-carotene in serum and thiamine diphosphate (TDP) in whole blood were determined by HPLC. Retinol <1.05 µmol/L was considered indicative of low vitamin A and TDP <65 nmol/L of deficient thiamine status [21,22]. Serum cholesterol and triglycerides were analyzed by enzymatic methods; total serum fat (to adjust α-tocopherol) was estimated using an equation as proposed by Rylander *et al.* [23]: total lipids (g/L) = 0.92 + 1.31 × (cholesterol + triglycerides).

Whole blood samples were transferred to the Institute of Nutrition, Jena, to analyze 5-methyltetrahydrofolate (5-MTHF) by HPLC as previously described [24] with modifications: Samples were incubated with dithiothreitol (20 g/L) in addition to ascorbic acid solution (20 g/L), and an Eurospher II analytical column (100-5 Phenyl 250 × 4.6 mm, Knauer GmbH, Germany) in combination with a mobile phase consisting 17.5% methanol (*v*/*v*) in formic acid buffer (70 mmol/L, pH 3.75) was used for the separation of 5-MTHF. The method allowed the simultaneous analysis of tryptophan, which was quantified against a pure standard (L-Tryptophan, Sigma-Aldrich, Steinheim, Germany).

### 2.5. Statistical Analysis

Maternal characteristics and data on blood MNs were described using means ± SD, frequencies (%) and medians with interquartile range. Blood MNs and MN deficiencies were compared between surveys (2006 *vs.* 2004) and for corresponding trimesters using the Student′s *t*-test, the Mann-Whitney U test, or Chi-squared analysis, as appropriate. For comparisons across trimesters within the same study year, ANOVA, Kruskal-Wallis and Chi-squared tests were used.

Factors associated with blood MNs in pregnancy were identified using backward stepwise linear regression (*p* ≤ 0.05 to remain in model) using each blood MN as the dependent variable (distributions transformed to normal, where necessary). Random effects were fitted for trimester of blood draw and study year. Additional covariates considered included: own garden (growing fruit/vegetables), own chicken or ducks, own pigs or goats, daily betel, daily use of fish paste, and elevated inflammation markers (CRP > 5 mg/L, AGP > 1 g/L). The weeks of antenatal supplementation of iron, thiamine and folic acid were included in the models on iron status parameters (ferritin, sTfR), TDP and 5-MTHF. Total serum fat was included in the model on α-tocopherol. All models were adjusted for parity (0/1+), smoking status, and BMI.

## 3. Results

In June 2004, 533 out of 764 women (70%) participated in the first survey, and, in November 2006, 515 out of 745 (69%) registered pregnant women in the SMRU antenatal clinics participated in the second cross-sectional study (Table 1). The main reasons for the difference between registered and enrolled women were absence on the day of the cross-sectional survey (156/1509, 10%) and non-receipt of the refugee food ration (156/1509, 10%).

Years of residence in Maela and weeks of provided MN supplements at blood draw were higher in 2006 than in the 2004 survey. Results from the questionnaires at study enrollment revealed similar proportions of women who smoked and ate fermented fish paste 2–3 times daily, but a higher proportion of women in 2006 who chewed regularly (daily) betel nut; the proportion of households with their own garden (growing fruits/vegetables), chickens or ducks decreased, whereas more women reported raising either pigs or goats in 2006 than in 2004. Poultry in the camp were culled prior to the 2006 survey, due to the Asian bird flu outbreak (H5N1) [25].

MFF as an additional food ration in the basic food basket increased estimated daily intakes, in particular for vitamin A, B-vitamins (thiamine, riboflavin and nicotinamide), ascorbic acid, zinc and iron, while the supplementary oil ration for pregnant women especially improved the intakes of tocopherols (Table 2). In both surveys, provided iron, folate and thiamine covered several fold (2 to >50-fold), the recommended daily intakes for pregnant women.

**Table 1:** Demographics of pregnant women at the time of the cross-sectional survey.

Characteristics	2004 (*n* = 533)	2006 (*n* = 515)	*p*
Age (years) ^1^	26.4 ± 6.3	27.0 ± 7.2	0.173
Years in Thailand (years) ^2^	10.0 (5, 15)	10.0 (6, 16) *	0.471
Years in Maela (years)	7.0 (4, 9)	8.0 (5, 11) *	<0.001
Weeks of MN suppl. (weeks)	12.0 (5, 18)	14.0 (6, 21)	0.001
Religion, *n* (%) ^3^			
Buddhist	226 (42.4)	243 (47.2)	
Muslim	90 (16.9)	84 (16.3)	0.378
Christian	216 (40.5)	186 (36.1)	(3 d*f*)
Other/unknown	1 (0.2)	2 (0.4)	
Smoker, *n* (%)	150 (28.1)	138 (26.8)	0.625
Daily use of betel nut, *n* (%)	105 (19.7)	142 (27.7) *	0.002
Daily use of fermented fish-paste, *n* (%)	302 (56.7)	281 (54.8) *	0.540
Own garden (fruits/vegetables), *n* (%)	248 (46.5)	203 (39.6) *	0.023
Own chickens or ducks, *n* (%)	106 (19.9)	32 (6.2) *	<0.001
Own pigs or goats, *n* (%)	95 (17.8)	157 (30.6) *	<0.001
Parity, *n* (%)			
0	110 (20.6)	134 (26.0)	
1	135 (25.3)	100 (19.4)	0.025
≥2	288 (54.0)	281 (54.6)	(2 d*f*)
Trimester at time of x-sectional survey			
1st trimester, *n* (%)	89 (16.7)	64 (12.4)	
2nd trimester, *n* (%)	249 (46.7)	225 (43.7)	0.026
3rd trimester, *n* (%)	195 (36.6)	226 (43.9)	(2 d*f*)
Height (cm)	151.2 ± 5.3	150.9 ± 5.5	0.370
Weight, if 1st trimester (kg)	49.3 ± 7.6	47.5 ± 7.4	0.151
Weight, if 2nd trimester (kg)	51.0 ± 6.9	51.5 ± 7.7	0.485
Weight, if 3rd trimester (kg)	54.8 ± 6.9	55.7 ± 8.1	0.222
BMI, if 1st trimester (kg/m^2^)	21.6 ± 3.0	20.9 ± 2.6	0.143
BMI, if 2nd trimester (kg/m^2^)	22.2 ± 2.8	22.5 ± 2.9	0.292
BMI, if 3rd trimester (kg/m^2^)	24.0 ± 2.8	24.4 ± 3.0	0.149
BMI < 18.5 kg/m^2^, *n* (%) ^4^	24 (4.5)	19 (3.7)	
BMI ≥ 18.5 to 23 kg/m^2^, *n* (%) ^4^	280 (52.5)	241 (46.8)	0.180
BMI ≥ 23 to 27.5 kg/m^2^, *n* (%) ^4^	192 (36.0)	209 (40.6)	(3 d*f*)
BMI ≥ 27.5 kg/m^2^, *n* (%) ^4^	37 (6.9)	46 (8.9)	

^1^ Values are mean ± SD; ^2^ median (IQR, interquartile range); ^3^ and number (%); ^4^ cautious interpretation required as the proportion of BMI categories groups are not controlled for trimester; * missing values for *n* = 2 women. MN, micronutrient; BMI, body mass index.

**Figure A1:**
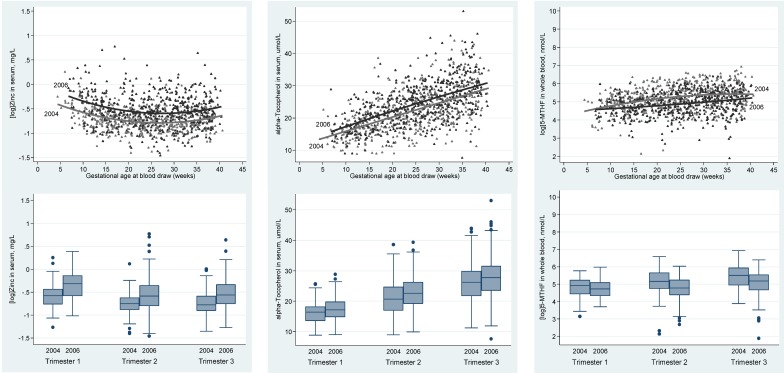
Serum zinc, α-tocopherol, and whole blood 5-methyltetrahydrofolate (5-MHTF) across gestation and by study year.

Significant changes across gestation, from trimester 1 to 3, were observed for most of the measurements including hemoglobin, markers of inflammation and MNs (Table 3, Figure A1). A significant decrease in hemoglobin, ferritin, zinc, retinol and tryptophan from first to third trimester were accompanied by a significant increase in soluble transferrin receptor (sTfR), copper, cholesterol, α/γ-tocopherol, 5-MHTF and TDP (Table 3). Notably, serum fat-adjusted α-tocopherol did not vary between trimesters. There was less power to show a difference in trimester 1 due to smaller numbers. Nevertheless, the comparison between surveys showed in at least two consecutive trimesters in 2006 significantly higher zinc, copper, α-tocopherol, fat-corrected α-tocopherol, α-carotene and tryptophan, and significantly lower retinol and 5-MHTF than in 2004.

**Table 2:** Estimated daily micronutrient intake by provided food rations and supplements for pregnant women in 2006 *vs.* 2004.

Micronutrient ^1^	Basic Food ^2^ Basket 04	Total Food ^3^ June 2004	Basic Food ^2^ Basket 06	Total Food ^3^ November 2006	RDI ^4^
Vitamin A, µg RE ^1^ (%)	158 (20)	179 (22)	390 (49)	504 (63)	800
α-TEs ^1^, mg (%)	8.5 (57)	10.0 (67)	8.6 (57)	15.2 (101)	15
Thiamine, mg (%)	0.7 (50)	1.2 (83)	1.1 (78)	1.4 (100)	1.4
Riboflavin, mg (%)	0.4 (29)	0.7 (47)	1.1 (81)	1.4 (99)	1.4
Nicotinamide, mg (%)	10.3 (57)	14.0 (78)	12.5 (70)	19.0 (105)	18
Folic acid, µg (%)	334 (56)	715 (119)	326 (54)	668 (111)	600
Cobalamin, µg (%)	1.1 (42)	3.3 (126)	1.4 (53)	3.1 (118)	2.6
Ascorbic acid, mg (%)	5.6 (10)	13.6 (25)	26.2 (48)	29.2 (53)	55
Zinc, mg (%)	7.6 (76)	9.9 (99)	9.9 (99)	13.1 (131)	10
Copper, mg (%)	1.7 (174)	2.5 (248)	1.5 (149)	2.6 (259)	1.0
Iron, mg (%)	9.0 (33)	14.7 (54)	16.2 (60)	22.0 (82)	27
Calcium, mg (%)	138 (12)	263 (22)	183 (15)	336 (28)	1200
***Supplements***					
Thiamine, mg (%)		92 (6571)		92 (6571)	1.4
Folic acid, µg (%)		5000 (833)		5000 (833)	600
Iron, mg (%)		221 (221)		221 (221)	100

^1^ RE, Retinol equivalents (1 RE = 1 µg retinol or 12 µg beta-carotene); α-TEs, α-Tocopherol Equivalents (1 mg α-TE = 1 mg d-α-tocopherol or 2 mg d-β-tocopherol or 10 mg d-γ-tocopherol or 3.3 mg d-α-tocotrienol or 33.3 mg d-δ-tocopherol). Food values were taken from the USDA National Nutrient Database for Standard Reference [26] and Souci, Fachmann, Kraut [27]; ^2^ basic monthly food basket in June 2004 contained 16 kg (polished) rice, 1.5 kg mung beans, 1 kg fermented fish, 1 L soybean oil and 125 g of dried chilies; in November 2006, the monthly food basket for adults contained additionally 1.4 kg of micronutrient fortified flour (MFF) but a lower amount of rice (15 kg) and split mung beans (1 kg); ^3^ Total food in 2004 consisted of the basic food basket and the weekly supplementary food rations (500 g split mung beans and 300 g dried fish) for pregnant women. Total food in 2006 contained the basic food basket (including MFF) and the weekly supplementary rations of red beans (500 g) tinned fish (310 g) and additionally provided soybean oil (250 mL). Daily provided MN supplements consisted of 600 g ferrous sulfate (221 g elemental iron), 5 mg folic acid and 100 mg thiamine mononitrate (92 mg thiamine). MFF, micronutrient fortified flour, which includes a MN premix and MNs given for rice flour (75%) and soya flour (25%); ^4^ recommended daily intakes (RDI) for pregnant women (not specific for gestational age) as suggested by FAO/WHO and the Institute of Medicine (IOM), USA (α-TE, iron) [28].

**Table 3:** Comparison of hemoglobin, blood MNs and nutritional markers between surveys and across trimesters after grouping by trimester of blood draw ^1^.

Nutritional Markers	Tr	*n*	2004	*n*	2006	*p*
Hemoglobin (g/L)	1	89	115.9 ± 11.0	64	113.7 ± 10.0	0.209
	2	249	107.5 ± 11.1	223	107.8 ± 11.2	0.758
	3	195	109.0 ± 11.8	226	107.9 ± 11.6	0.356
						
(trimester trend)	*P*		<0.001		<0.001	
CRP > 5 mg/L, *n* (%)	1	88	20 (22.7)	64	9 (14.1)	0.179
	2	248	49 (19.8)	225	49 (21.8)	0.588
	3	195	33 (16.9)	226	40 (17.7)	0.834
**	*P*		0.459		0.303	
AGP > 1 g/L, *n* (%)	1	88	9 (10.2)	64	8 (12.5)	0.661
	2	248	7 (2.8)	225	1 (0.4)	0.045
	3	195	2 (1.0)	226	2 (0.9)	0.882
**	*P*		<0.001		<0.001	
Ferritin (µg/L)	1	88	57.0 (33.0, 80.6)	64	70.1 (49.4, 108.6)	0.023
	2	248	39.5 (20.3, 68.1)	225	39.9 (22.0, 64.3)	0.923
	3	195	30.2 (15.2, 49.8)	226	24.7 (12.5, 43.8)	0.034
**	*P*		<0.001		<0.001	
sTfR (mg/L)	1	88	5.81 (4.50, 7.05)	64	5.52 (4.72, 6.63)	0.811
	2	248	5.96 (4.88, 7.55)	225	6.04 (5.16, 7.46)	0.284
	3	195	7.27 (5.70, 8.99)	226	7.25 (5.97, 8.80)	0.456
**	*P*		<0.001		<0.001	
Zinc (mg/L)	1	88	0.560 (0.47, 0.65)	64	0.726 (0.54, 0.87)	<0.001
	2	248	0.471 (0.41, 0.54)	225	0.558 (0.45, 0.70)	<0.001
	3	195	0.460 (0.40, 0.55)	226	0.571 (0.47, 0.71)	<0.001
**	*P*		<0.001		<0.001	
Copper (mg/L)	1	88	1.61 (1.40, 1.85)	64	1.68 (1.45, 2.02)	0.307
	2	248	2.07 (1.82, 2.31)	225	2.22 (1.87, 2.65)	<0.001
	3	195	2.14 (1.87, 2.41)	226	2.28 (1.95, 2.80)	<0.001
**	*P*		<0.001		<0.001	
Cholesterol (mmol/L)	1	88	3.80 ± 0.66	64	4.03 ± 0.73	0.041
	2	248	4.66 ± 1.03	225	4.95 ± 1.03	0.002
	3	195	5.73 ± 1.17	226	5.95 ± 1.32	0.071
**	*P*		<0.001		<0.001	
Triglycerides (mmol/L)	1	88	1.16 (0.90, 1.52)	64	1.20 (0.91, 1.55)	0.690
	2	248	2.05 (1.55, 2.57)	225	2.07 (1.65, 2.75)	0.176
	3	195	2.93 (2.36, 3.67)	226	3.10 (2.38, 3.87)	0.325
**	*P*		<0.001		<0.001	
α-Tocopherol (µmol/l)	1	88	16.19 ± 3.54	64	17.60 ± 4.28	0.028
**	2	248	20.86 ± 5.49	225	22.85 ± 5.25	<0.001
**	3	195	26.25 ± 6.34	226	28.06 ± 6.73	0.005
**	*P*		<0.001		<0.001	
α-Tocopherol/fat (µmol/g)	1	88	3.17 ± 0.47	64	3.33 ± 0.61	0.073
**	2	248	3.13 ± 0.55	225	3.26 ± 0.60	0.012
**	3	195	3.09 ± 0.54	226	3.21 ± 0.56	0.027
**	*P*		0.505		0.327	
γ-Tocopherol (µmol/L)	1	88	2.26 (1.55, 3.07)	64	2.19 (1.56, 2.96)	0.528
	2	248	2.54 (1.73, 3.41)	225	2.61 (1.78, 3.45)	0.730
	3	195	2.90 (2.04, 3.73)	226	2.60 (1.98, 3.70)	0.332
**	*P*		0.001		0.003	
Retinol (µmol/L)	1	88	1.40 ± 0.33	64	1.28 ± 0.34	0.042
	2	248	1.48 ± 0.41	225	1.35 ± 0.32	<0.001
	3	195	1.35 ± 0.40	226	1.25 ± 0.39	0.013
**	*P*		0.001		0.016	
α-Carotene (µmol/L)	1	88	0.040 (0.02, 0.06)	64	0.064 (0.04, 0.10)	<0.001
	2	248	0.042 (0.03, 0.07)	225	0.069 (0.05, 0.11)	<0.001
	3	195	0.041 (0.03, 0.06)	226	0.071 (0.05, 0.11)	<0.001
**	*P*		0.294		0.774	
β-Carotene (µmol/L)	1	88	0.267 (0.18, 0.40)	64	0.200 (0.17, 0.32)	0.060
	2	248	0.249 (0.16, 0.37)	225	0.221 (0.16, 0.31)	0.052
	3	195	0.216 (0.13, 0.32)	226	0.216 (0.14, 0.31)	0.946
**	*P*		0.035		0.875	
TDP (nmol/L)	1	89	96.96 (75.15, 119.4)	63	107.7 (84.93, 131.9)	0.062
	2	249	109.6 (83.96, 140.5)	225	108.6 (87.42, 139.7)	0.890
	3	195	111.0 (83.99, 145.5)	226	110.8 (79.36, 139.2)	0.246
**	*P*		0.009		0.842	
5-MTHF (nmol/L)	1	89	138.4 (83.72, 190.6)	63	112.4 (72.44, 162.9)	0.165
	2	248	174.3 (115.3, 284.9)	225	119.2 (79.72, 190.1)	<0.001
	3	195	246.2 (142.2, 376.0)	226	178.2 (108.5, 252.5)	<0.001
**	*P*		<0.001		<0.001	
Tryptophan (µmol/L)	1	89	27.96 ± 5.71	63	28.53 ± 8.46	0.621
	2	248	25.72 ± 6.86	225	28.07 ± 5.98	<0.001
	3	195	24.20 ± 5.58	226	26.51 ± 4.98	<0.001
**	*P*		<0.001		0.006	

^1^ *p*: surveys compared by Student′s *t*-test, Chi-squared test and Mann Whitney-U test; *P*: trimester trend assessed by ANOVA, Chi-squared test and Kruskall-wallis test. CRP, C-reactive protein; AGP, α-1 glycoprotein; sTfR, soluble transferrin receptor; TDP, thiamine diphosphate; 5-MTHF, 5-methyltetrahydrofolate.

The mean hemoglobin and the high prevalence of anemia (60%) and iron deficiency (38.5%) in the third trimester remained constant (Table 4), despite MFF and the provision of iron supplements.

**Table 4:** Comparison of the prevalence of anemia and MN deficiencies between surveys ^1^.

Anemia, MN Deficiencies	Tr	*n*	2004	*n*	2006	*p*
Anemia, *n* (%)	1	89	29 (32.6)	64	24 (37.5)	0.528
	2	249	93 (37.3)	223	79 (35.4)	0.665
	3	195	118 (60.5)	226	134 (59.3)	0.799
(trimester trend)	*P*		<0.001		<0.001	
Iron deficiency, *n* (%)	1	88	13 (14.8)	64	6 (9.4)	0.320
	2	248	57 (23.0)	225	39 (17.3)	0.127
	3	195	75 (38.5)	226	87 (38.5)	0.994
**	*P*		<0.001		<0.001	
Zinc deficiency, *n* (%)	1	88	44 (50.0)	64	17 (26.6)	0.004
	2	248	164 (66.1)	225	73 (32.4)	<0.001
	3	195	117 (60.0)	226	69 (30.5)	<0.001
**	*P*		0.026		0.660	
Retinol <1.05 µmol/L, *n* (%)	1	88	15 (17.0)	64	15 (23.4)	0.328
	2	248	33 (13.3)	225	39 (17.3)	0.223
	3	195	44 (22.6)	226	75 (33.2)	0.016
**	*P*		0.038		0.001	
TDP <65 nmol/L, *n* (%)	1	89	14 (15.7)	63	6 (9.5)	0.265
	2	249	27 (10.8)	225	17 (7.6)	0.218
	3	195	16 (8.2)	226	28 (12.4)	0.162
**	*P*		0.162		0.228	

^1^ *p*: surveys compared by Student′s *t*-test, Chi-squared test and Mann Whitney-U test.

Modifiable determinants associated with a significant positive effect on micronutrients included consumption of fish paste, ownership of farm animals (pigs/goats or chicken/ducks) and a garden (fruit/vegetables), and increased weeks of micronutrient supplements (Table 5). The daily consumption of betel nut was inversely associated with hemoglobin and 5-MTHF. Blood samples with elevated infection markers (CRP > 5 mg/L and/or AGP > 1 g/L) were associated with a higher mean ferritin but lower mean retinol, α-/β-carotene and tryptophan (Table 5). As zinc was not associated with any determinants in the model, this suggests that increases in this MN between 2004 and 2006 were due to the MFF.

**Table 5:** Determinants of hemoglobin and micronutrient status marker in pregnancy ^1^.

MN Status Marker	Beta	95% CI
**Hemoglobin**, g/L		
Betel	−2.90	−4.62 to −1.16
(log) **Ferritin**, µg/L		
AGP > 1 g/L	0.320	0.002 to 0.637
CRP > 5 mg/L	0.201	0.068 to 0.334
Fish paste (daily)	0.141	0.038 to 0.244
MN suppl., weeks	−0.022	−0.028 to −0.016
(log) **sTfR**, mg/L		
Owns pigs or goats	−0.048	−0.094 to −0.002
MN suppl., weeks	0.008	0.005 to 0.010
(log) **Zinc**, mg/L	-	-
**Copper**, mg/L		
CRP > 5 mg/L	0.329	0.248 to 0.409
**α-Tocopherol**, µmol/L		
Owns pigs or goats	0.582	0.005 to 1.16
AGP > 1 g/L	−2.28	−3.83 to −0.733
CRP > 5 mg/L	1.30	0.656 to 1.95
Total serum fat, g/L	2.79	2.65 to 2.93
Fish paste (daily)	−0.908	−1.41 to −0.405
(log) **γ-Tocopherol**, µmol/L		
AGP > 1g/L	−0.287	−0.474 to −0.101
CRP > 5mg/L	0.144	0.065 to 0.222
Fish paste (daily)	−0.074	−0.134 to −0.013
**Retinol**, µmol/L		
AGP > 1 g/L	−0.288	−0.429 to −0.147
CRP > 5 mg/L	−0.113	−0.173 to −0.054
Owns chickens or ducks	0.075	0.008 to 0.141
Fish paste (daily)	0.069	0.024 to 0.115
(log) **α-carotene**, µmol/L		
Betel (daily)	0.765	0.608 to 0.923
CRP > 5 mg/L	−0.249	−0.414 to −0.084
AGP > 1 g/L	−0.886	−1.28 to −0.495
Owns chickens or ducks	−0.202	−0.386 to −0.017
Owns pigs or goats	0.280	0.134 to 0.425
(log) **β-carotene**, µmol/L		
AGP > 1 g/L	−0.422	−0.670 to −0.174
CRP > 5 mg/L	−0.117	−0.221 to −0.012
Own garden	0.116	0.036 to 0.196
Fish paste (daily)	0.106	0.025 to 0.186
**TDP**, nmol/L		
MN suppl., weeks	0.436	0.144 to 0.729
(log) **5-MTHF**, nmol/L		
MN suppl., weeks	0.022	0.017 to 0.027
Own garden	0.090	0.008 to 0.171
Betel (daily)	−0.133	-0.234 to −0.031
**Tryptophan**, µmol/L		
Owns pigs or goats	0.920	0.040 to 1.80
CRP > 5 mg/L	−1.19	−2.18 to −0.203
AGP > 1 g/L	−3.93	−6.29 to −1.58

^1^ All models were adjusted for smoking (0/1), parity (0/1+) and BMI at the time of sampling; variables retained in the table were all significant at *p* < 0.05.

## 4. Discussion

The introduction of MFF and the additional supplementary oil ration did not result in a uniform improvement in MN status in pregnant mothers: Serum zinc and α-tocopherol increased in each trimester, but 5-MTHF and retinol were lower in the second survey. The improved zinc status in a population with predominantly plant-based and phytate-rich staple food (mung beans) as the main source of zinc is most likely attributed to zinc from the MFF, and this was also observed in lactating mothers in the Maela camp with the introduction of MFF [5]. Higher-serum α-tocopherol in each trimester is very likely due to the additional supplementary ration of soybean oil. The increase in α- but not in γ-tocopherol, despite the high content of γ-tocopherol in soybean oil, could be explained by the accumulation of γ-tocopherol in tissues (skin, muscle, adipose) and its relatively rapid conversion to the water-soluble metabolite γ-CEHC [29,30]. Lower retinol and β-carotene in 2006 compared to 2004 may be due to the lower proportion of households with their own chicken/ducks and own garden, and to the timing of the first survey, which coincided with mango season, providing a rich source of β-carotene. The lower 5-MTHF in the second cohort may have resulted from a higher vitamin B_12_ intake through the flour which could result in a higher activity of methionine synthase and thus a higher metabolism rate of 5-MTHF [31]. A decrease in compliance to the supplement in the second survey seems unlikely given the significant increase in 5-MTHF (and TDP) with the number of weeks of folic acid (and thiamine) supplementation in both surveys. The study has confirmed that hemoglobin, iron status and several blood MNs including zinc, retinol, and tryptophan decrease at different stages of pregnancy, while α-/γ-tocopherol and copper steadily increase, which is in agreement with studies in both Western and developing countries [6,10,13,20,32,33,34]. The variation in hemoglobin and blood MN status with advanced gestation can be explained by increased erythropoiesis, high requirements of MNs for fetal growth, physiological hemodilution and the hyperlipidemic state in pregnancy [35,36].

MFF as a new food ration might have been responsible for the slightly higher first-trimester ferritin in the second cohort. Nevertheless, the high prevalence of anemia (60%) and iron deficiency (38.5%) in the third trimester, despite MFF and the provision of iron supplements, suggests low compliance or coexisting helminth infections, which has been found in this population previously [37]. Inadequate riboflavin intake increased the risk of anemia in Chinese adults [38], and riboflavin and retinol, given along with iron and folic acid, was more effective in reducing anemia and iron deficiency in pregnant women than iron and folic acid alone [39]. In a follow-up study in India, iron supplementation (60 mg) combined with a deworming- and nutritional education program increased serum ferritin and hemoglobin, and significantly reduced the prevalence of anemia in each trimester [40]. However, the mean 3rd trimester serum ferritin (25 µg/L) and hemoglobin (105 g/L) in the iron-supplemented women were similar to those in the Maela camp. The decrease in iron status (low ferritin/high sTfR) and hemoglobin, particularly in the 2nd trimester of pregnancy, were also reported in iron-supplemented Indonesian and Vietnamese women [41,42]. In this context, the significantly higher retinol and serum ferritin in those women who frequently consumed fish paste, as well as the improvement of first-trimester ferritin in those who were provided with MFF, are important findings of the present study. A previous study in the same population did not find a relationship between anemia (measured by hematocrit) at the first antenatal visit and betel nut consumption [43], but the model presented here suggests betel nut consumption has a strong negative impact on hemoglobin; this identifies a potentially large (due to the large proportion of betel nut users) modifiable risk factor for anemia. The negative impact by betel nut could be explained by its effect to lower TDP (thiaminases in the areca nut) and other vitamins such as folate and cobalamin [44] involved in the erythropoiesis. Both TDP and 5-MTHF were lower in women who chewed betel, and were significantly and positively associated with hemoglobin in the present study.

The significantly higher α-carotene in betel nut chewers is most likely due to α-carotene present in the leaves of *Piper betel* L. [45], which are used, sometimes with slaked lime, to wrap small pieces of areca nut to form the “betel quid” [43]. Higher β-carotene and 5-MTHF in women who reported garden ownership, and higher ferritin, retinol and β-carotene in those who regularly consumed fish paste, highlights gardens and MN-rich staple foods (e.g., fish paste, MFF) as strategies for preventing MN deficiencies in protracted refugee settings [46]. The women who reported owning pigs/goats or chicken/ducks were associated with less tissue iron deficiency (inverse association with sTfR) and with a higher retinol, α-tocopherol, β-carotene and tryptophan. Higher mean tryptophan (TRP) in the second cohort might be due to an increased niacin intake through the fortified flour and therefore lower demand of TRP for the translation into niacin [47,48].

The limitations of the present study include only a coarse assessment of nutritional habits and the lack of detailed data regarding compliance to the supplements. However, the assessment of dietary intake was not the main focus of the project and the dietary variety was very restrictive and mainly based on the acceptance of the provided food and MN supplements. Unfortunately, all the MNs present in the flour were not measured.

## 5. Conclusions

In conclusion, hemoglobin, iron status and circulating blood MNs were associated with food rations, MN supplements, season and habits, as well the trimester of blood draw. Thiamine (TDP) and folate (5-MTHF) in blood indicated a successful supplementation regimen, whereas the high prevalence of iron deficiency in late pregnancy suggests a low compliance to ferrous sulfate and possibly further factors (e.g., helminth infections, coexisting MN deficiencies) negatively affecting iron metabolism. MFF as a new staple food and the additional oil ration for pregnant women improved zinc and α-tocopherol status, respectively. Multiple MN supplements, MN-rich staple food, small gardens and farm animals seem to be promising strategies for reducing the prevalence of MN deficiencies during pregnancy in protracted refugee settings. Programs that improve iron compliance and reduce betel nut consumption are required.

## Appendix

See end of the manuscript: Figure A1. Serum zinc, α-tocopherol, and whole blood 5-methyltetrahydrofolate (5-MHTF) across gestation and by study year.
